# On the Use of Turf as Bedding

**Published:** 1896-08

**Authors:** 


On the Use of Turf as Bedding. On account of the dearth of straw during the year 1893 the authorities of the central depot of Swiss cavalry decided to utilize turf as bedding. During a year and a half turf was used for five hundred horses. The preference was given to Dutch turf, containing long vegetable fibres, having a clear color and being as dry as possible. This turf (or peat) contains about 20 per cent. of humidity, 1.5 per cent. of ashes, and a little azote. The bed should be the object of much care, and the dung should be removed as often as possible; at first about 80 kilos of turf were put in for five weeks; daily 2.5 kilos of dry turf were added, so that the whole bed was renewed at the end of six weeks. Each horse used, therefore, 160 kilos, or 2.5 kilos per day; in estimating the cost of 100 kilos at 3.80 francs, the cost of bedding is 10 centimes a day for each horse. It goes without saying that these figures depend upon the care taken in the expenditure of bedding, the price of turf, etc. The chief advantage resulting from the use of turf consists in its great power of absorption for liquids and ammonium salts. When it is dry it absorbs about 70 per cent. of liquid, or double as much as straw. The disinfectant qualities of this bedding can no longer be denied; the author believes he can attribute to the use of this bedding the diminution in the number of cases of pneumonia occurring in the depot during its use. As an offset to these advantages M. Schwendimann mentions some disadvantages. The hoofs soften and become brittle, the sole too flexible and sensitive, the frog rots; these effects are due to the action of urine and ammonia, two destroyers of horn. On the other hand, the horses placed upon turf without straw could not eat, grew languid, learned to be vicious or to tease their neighbors. These were the reasons which led the authorities to discontinue its use. M. Schwendimann is of the opinion that a mixed under-bed of turf and a covering of straw on top could be utilized, as is practised in the cavalry school in Hanover.—*Schweizer Archiv*, March and April, 1895; *Annales de Med. Vet.*

